# Angiotensin II Type 1 Receptor-associated Protein Inhibits Angiotensin II-induced Insulin Resistance with Suppression of Oxidative Stress in Skeletal Muscle Tissue

**DOI:** 10.1038/s41598-018-21270-8

**Published:** 2018-02-12

**Authors:** Kohji Ohki, Hiromichi Wakui, Nozomu Kishio, Kengo Azushima, Kazushi Uneda, Sona Haku, Ryu Kobayashi, Kotaro Haruhara, Sho Kinguchi, Takahiro Yamaji, Takayuki Yamada, Shintaro Minegishi, Tomoaki Ishigami, Yoshiyuki Toya, Akio Yamashita, Kento Imajo, Atsushi Nakajima, Ikuma Kato, Kenichi Ohashi, Kouichi Tamura

**Affiliations:** 1 Department of Medical Science and Cardiorenal Medicine, Yokohama City University Graduate School of Medicine, Yokohama, Japan; 2 Cardiovascular and Metabolic Disorders Program, Duke-NUS Medical School, Singapore, Singapore; 3 Department of Molecular Biology, Yokohama City University Graduate School of Medicine, Yokohama, Japan; 4 Department of Gastroenterology and Hepatology, Yokohama City University Graduate School of Medicine, Yokohama, Japan; 5 Department of Molecular Pathology, Yokohama City University Graduate School of Medicine, Yokohama, Japan

## Abstract

Enhancement of AT1 receptor-associated protein (ATRAP) in adipose tissue improves high fat diet (HFD)-induced visceral obesity and insulin resistance, and suppresses adipose oxidative stress. However, HFD loading is not a direct stimulatory factor for AT1 receptor. In the present study, we investigated the effect of chronic, low-dose angiotensin II (Ang II) stimulation on glucose and lipid metabolism in mice and functional role of ATRAP. ATRAP expression was higher in adipose tissue (5–10-fold) and skeletal muscle tissue (approximately 1.6-fold) in ATRAP transgenic (TG) mice compared with wild-type (WT) mice. After Ang II infusion, insulin sensitivity was impaired in WT mice, but this response was suppressed in TG mice. Unexpectedly, Ang II infusion did not affect the adipose tissue profile in WT or TG mice. However, in skeletal muscle tissue, Ang II stimulus caused an increase in oxidative stress and activation of p38 MAPK, resulting in a decrease in glucose transporter type 4 expression in WT mice. These responses were suppressed in TG mice. Our study suggests that Ang II-induced insulin resistance is suppressed by increased ATRAP expression in skeletal muscle tissue. Hyperactivity of AT1 receptor could be related to formation of insulin resistance related to metabolic syndrome.

## Introduction

The inextricable relationship between cardiovascular diseases and metabolic disorders is associated with their pathophysiological progression^1–4^. Accumulating evidence suggests that the renin–angiotensin system plays a role in this association. Numerous studies have shown that the angiotensin II type 1 (AT1) receptor signalling pathway is involved in regulating glucose and lipid metabolism^5,6^. Excessive activation of this receptor exacerbates pathological conditions of metabolic disorders, including diet-induced obesity and insulin resistance^7–10^.

AT1 receptor-associated protein (ATRAP) directly binds to the AT1 receptor and functions as an endogenous inhibitor, which functionally suppresses hyperactivation of this receptor^11–13^. Recently, we generated ATRAP transgenic (TG) mice using a 5.4-kb adiponectin promoter^14^. We demonstrated that enhancement of ATRAP expression in adipose tissue improved diet-induced visceral obesity and insulin resistance, concomitant with suppression of oxidative stress and inflammation of adipose tissue^15^. However, high-fat diet loading, which was used in our study, was not a direct stimulatory factor for the AT1 receptor.

Therefore, we investigated the effect of continuous angiotensin II (Ang II) stimulation on glucose and lipid metabolism in mice and the functional role of ATRAP in this process. First, we analysed visceral ATRAP mRNA expression levels in TG mice prior to our Ang II administration experiment. Based on our results, we reconfirmed that ATRAP expression was approximately 10-fold higher in visceral white adipose tissue (WAT) and approximately five-fold higher in brown adipose tissue (BAT) compared with wild-type (WT) mice^14,15^. Additionally, we found that ATRAP expression was higher (approximately 1.6-fold) in soleus muscle tissue in TG mice than in WT mice. After administering low doses of Ang II for 2 weeks, insulin sensitivity was impaired in WT mice, but this response was suppressed in TG mice. Our analysis of WAT indicated that an Ang II stimulus had no effect on adipose oxidative stress, inflammation, or adipocyte differentiation marker levels in WT or TG mice. In contrast, our analysis of skeletal muscle tissue indicated that an Ang II stimulus caused an increase in oxidative stress, which exacerbated insulin resistance only in WT mice.

## Results

### Enhancement of ATRAP expression in adipose tissue and skeletal muscle tissue of TG mice

**Figure 1:**
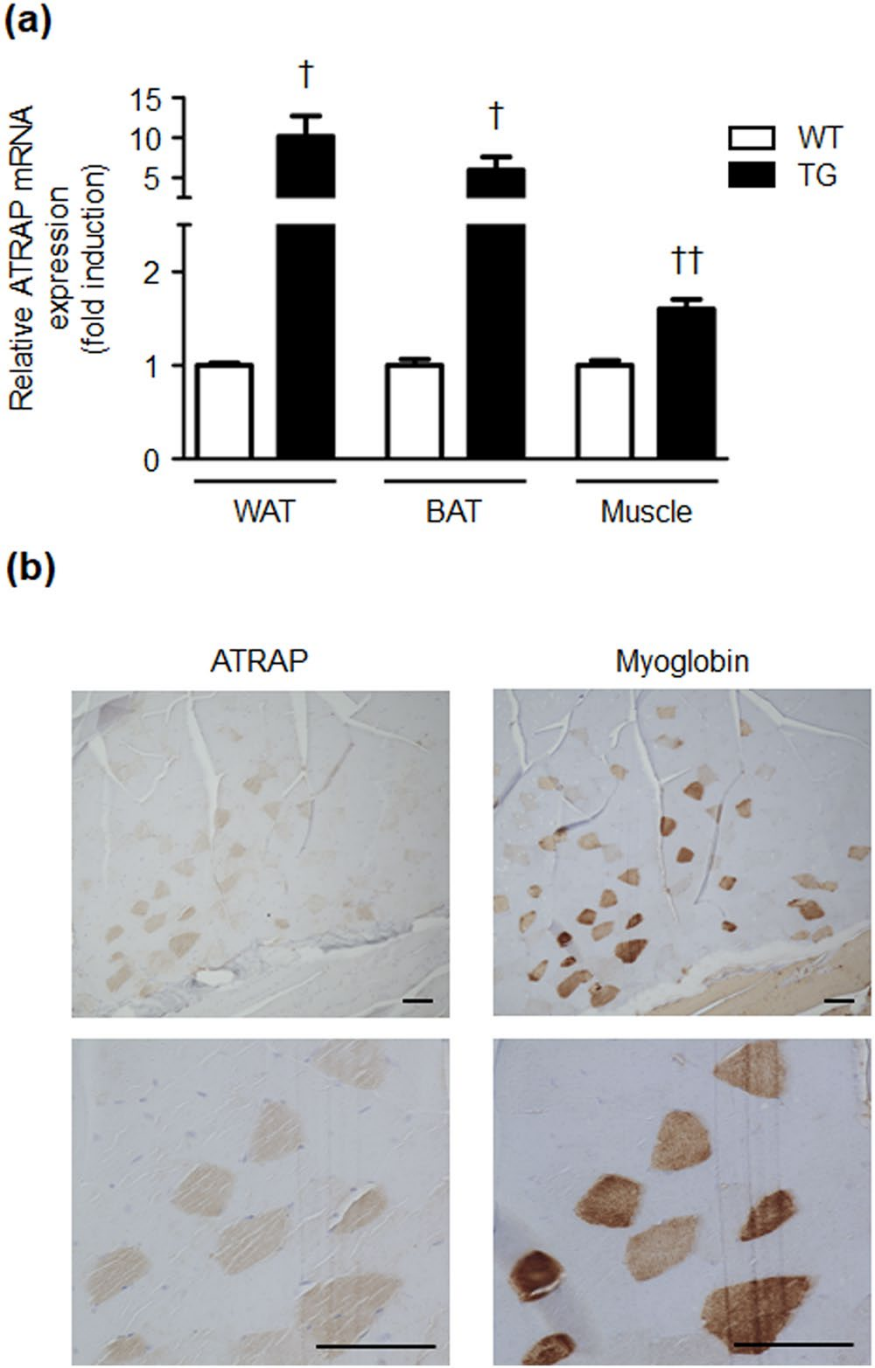
ATRAP expression in adipose tissue and skeletal muscle tissue of WT and TG mice. (**a**) Relative ATRAP mRNA levels in WAT, BAT, and skeletal muscle tissue from WT and TG mice. Values are expressed as mean ± SE (*n* = 3 in each group). ^†^*P* < 0.05, ^††^*P* < 0.01, vs WT mice. (**b**) Immunohistochemical analysis for ATRAP in skeletal muscle tissue of TG mice. The consecutive sections from the soleus muscle tissue of TG mice stained for ATRAP and myoglobin. Positive areas for ATRAP and myoglobin are shown as brown dots in each section. Original magnification, ×100 (upper panels), and ×400 (lower panels). Scale bar = 200 μm. WT, wild-type; TG, ATRAP transgenic mice; WAT, white adipose tissue; BAT, brown adipose tissue.

Recently we showed the tissue distribution of ATRAP expression in TG mice that were generated under control of the adiponectin promoter^15^. In this study, we reanalysed ATRAP mRNA expression in adipose tissue and skeletal muscle tissue of WT and TG mice. We reconfirmed that ATRAP mRNA expression in WAT and BAT of TG mice was markedly increased compared with WT mice (approximately 5–10-fold) (Fig. 1a). Additionally, fold induction analysis of mRNA levels in each tissue showed that ATRAP mRNA expression in skeletal muscle tissue of TG mice was slightly, but significantly, higher compared with WT mice (approximately 1.6-fold). To further investigate distribution of ATRAP in skeletal muscle tissue, we performed an immunohistochemical analysis using an anti-ATRAP antibody and a polyclonal antibody against myoglobin, which is specifically expressed in skeletal muscle cells. The results of consecutive sections stained with specific antibodies suggested that ATRAP was expressed in skeletal muscle cells (i.e., muscle fibres), but not other cells (e.g., macrophages and lymphocytes) in skeletal muscle tissue of TG mice (Fig. 1b).

### Effects of Ang II infusion on body weight, blood pressure, heart rate, and cardiac hypertrophy in WT and TG mice

**Figure 2:**
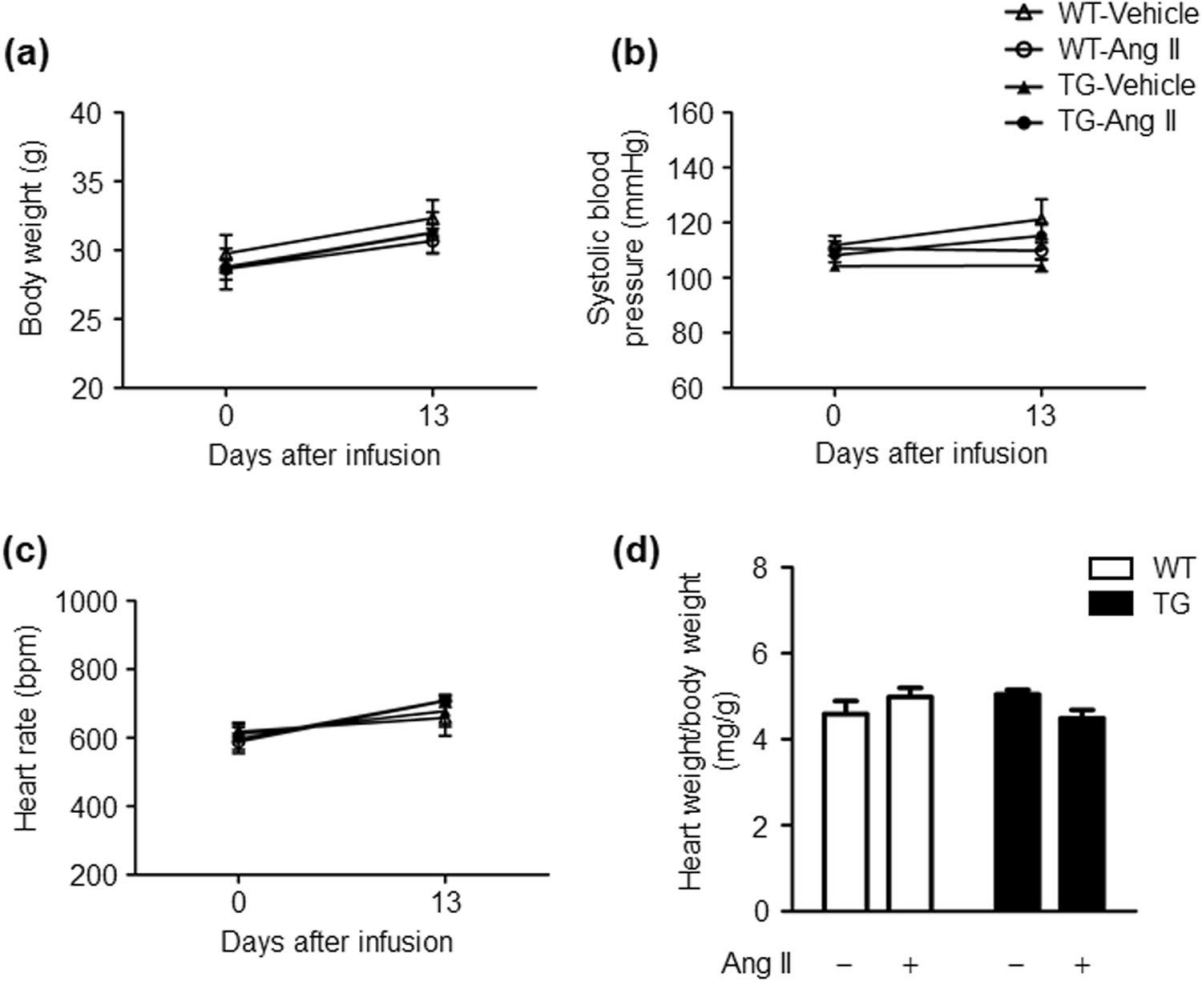
Ang II infusion does not affect body weight gain, blood pressure, heart rate, and the heart weight/body weight ratio in WT and TG mice. (**a**) Effects of Ang II infusion on body weight. (**b**) Effects of Ang II infusion on systolic blood pressure. (**c**) Effects of Ang II infusion on heart rate. (**d**) Effects of Ang II infusion on the heart weight/body weight ratio. Values are expressed as mean ± SE (*n* = 5 in each group). WT, wild-type; TG, ATRAP transgenic mice; Ang II, angiotensin II.

Age-matched WT and TG mice were divided into four groups: (1) vehicle-infused WT mice; (2) Ang II (100 ng/kg/min)-infused WT mice; (3) vehicle-infused TG mice; (4) Ang II (100 ng/kg/min)-infused TG mice. Ang II infusion did not affect body weight gain, systolic blood pressure and heart rate in WT and TG mice. There were also no differences in these parameters between the genotypes of vehicle or Ang II groups (Fig. 2a–c). We further examined the heart weight/body weight ratio in WT and TG mice because cardiac hypertrophy is closely associated with an elevation in blood pressure^16^. We found that the heart weight/body weight ratio was comparable in the vehicle- and Ang II-infused groups in WT and TG mice (Fig. 2d). Therefore, in this study, a 100 ng/kg/min dose of Ang II corresponded to a subpressor dose of Ang II.

### Effects of Ang II infusion on glucose and lipid metabolism in WT and TG mice

**Figure 3:**
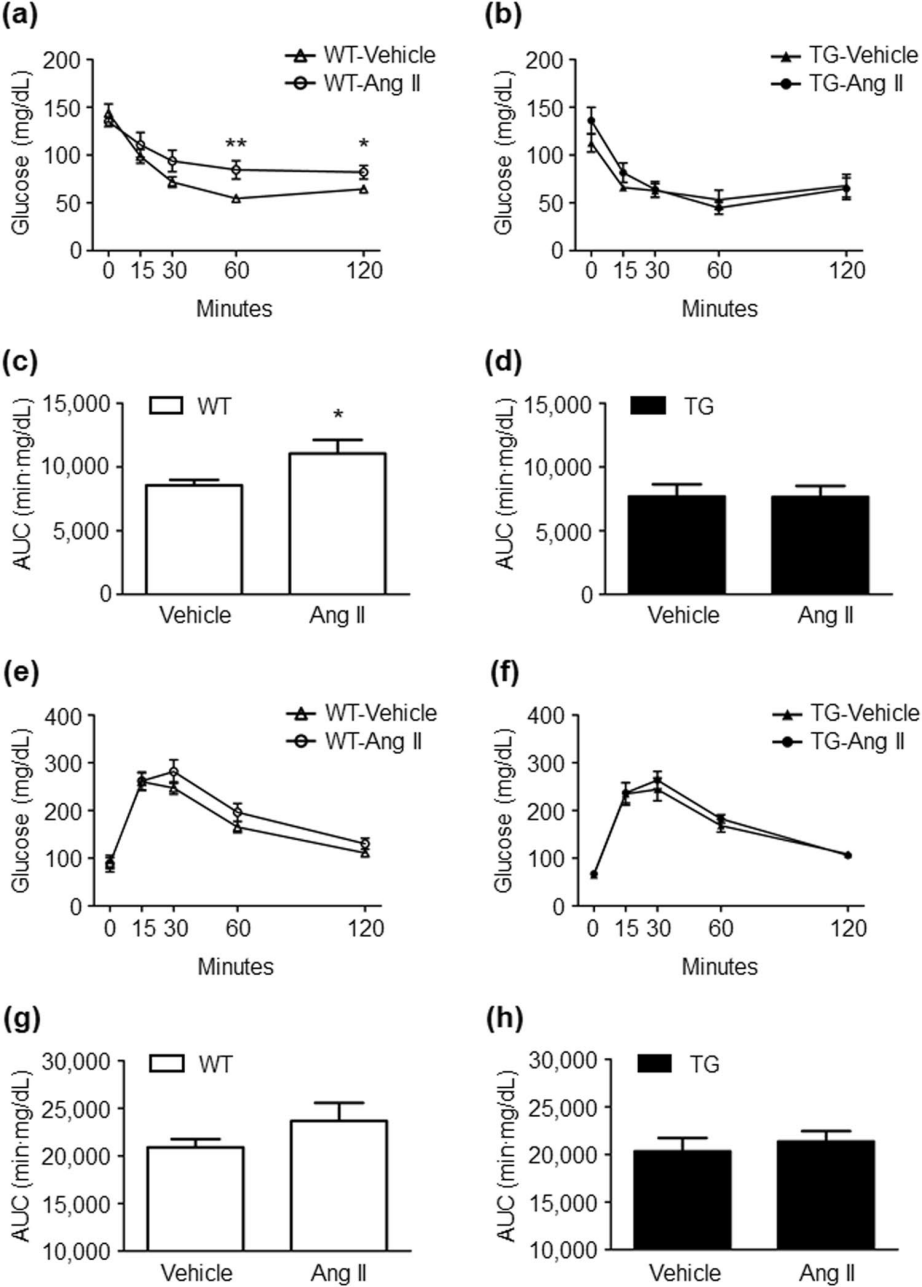
Ang II infusion induces insulin resistance and tends to impair glucose tolerance only in WT mice. The insulin tolerance test in WT (**a**) and TG mice (**b**), and the glucose tolerance test in WT (**e**) and TG mice (**f**) after 2 weeks of vehicle or Ang II infusion. The AUCs of the insulin tolerance test in WT (**c**) and TG mice (**d**), and those of the glucose tolerance test in WT (**g**) and TG mice (**h**) after 2 weeks of vehicle or Ang II infusion. Values are expressed as mean ± SE (*n* = 7–10 in each group). *P < 0.05, **P < 0.01, vs vehicle. WT, wild-type; TG, ATRAP transgenic mice; Ang II, angiotensin II; AUC, area under the curve.

We next examined the parameters of glucose and lipid metabolism using blood samples obtained by cardiac puncture at the time mice were sacrificed. Total cholesterol, triglyceride, free fatty acid, and non-fasting blood glucose levels were comparable in the vehicle- and Ang II-infused mice in both genotypes (Table 1). Although Ang II infusion significantly increased plasma insulin levels in WT mice, this increase in plasma insulin levels in response to Ang II infusion was suppressed in TG mice (Table 1). To further examine effects of Ang II infusion on insulin sensitivity, we performed the glucose tolerance test (GTT) and insulin tolerance test (ITT), which reflect insulin secretion and resistance, respectively (Fig. 3). Chronic Ang II infusion caused insulin resistance as estimated by the ITT in WT mice, but this response was suppressed in TG mice (vehicle vs Ang II in WT mice; at 60 minutes after insulin injection, 54.6 ± 3.7 vs 84.5 ± 9.6 mg/dl, *P = *0.006; at 120 minutes after insulin injection, 64.4 ± 3.0 vs 82.0 ± 7.2 mg/dl, *P = *0.022; area under the curve, 8562 ± 425 vs 11045 ± 1068 min × mg/dl, *P* = 0.027) (Fig. 3a–d). On the other hand, the results of GTT showed that Ang II infusion tended to cause impaired glucose tolerance in WT mice without statistical significance (vehicle vs Ang II; at 60 minutes after glucose injection, 165.1 ± 11.7 vs 196.0 ± 18.9 mg/dl, *P* = 0.095; at 120 minutes after glucose injection, 110.7 ± 6.1 vs 130.3 ± 11.6 mg/dl, *P = *0.081) (Fig. 3e–h). These results suggest that chronic Ang II infusion provokes insulin resistance only in WT mice, but not in TG mice.

**Table 1:** Parameters of lipid and glucose metabolism in WT and TG mice.

Plasma concentration	WT		TG	
	Vehicle	Ang II	Vehicle	Ang II
Total cholesterol (mg/dL)	80.0 ± 6.8	66.4 ± 3.6	77.8 ± 5.9	82.5 ± 4.9
Triglyceride (mg/dL)	20.5 ± 4.9	32.0 ± 9.0	22.8 ± 4.0	25.8 ± 6.1
Free fatty acid (µEq/L)	709 ± 136	652 ± 247	461 ± 247	1024 ± 225
Glucose (mg/dL)	223.8 ± 19.7	210.6 ± 18.1	227.8 ± 17.4	229.4 ± 13.8
Insulin (ng/mL)	0.37 ± 0.05	0.85 ± 0.06**	0.62 ± 0.06	0.60 ± 0.07

All of the values are mean ± SEM. **P < 0.01, vs the genotype-matched vehicle group. *n* = 5 per group. Data were analysed by one-way ANOVA. WT, wild-type; TG, ATRAP transgenic mice; Ang II, angiotensin II.

### Effects of angiotensin II infusion on adipose oxidative stress, adipokines and adipocyte differentiation in WT and TG mice

**Figure 4:**
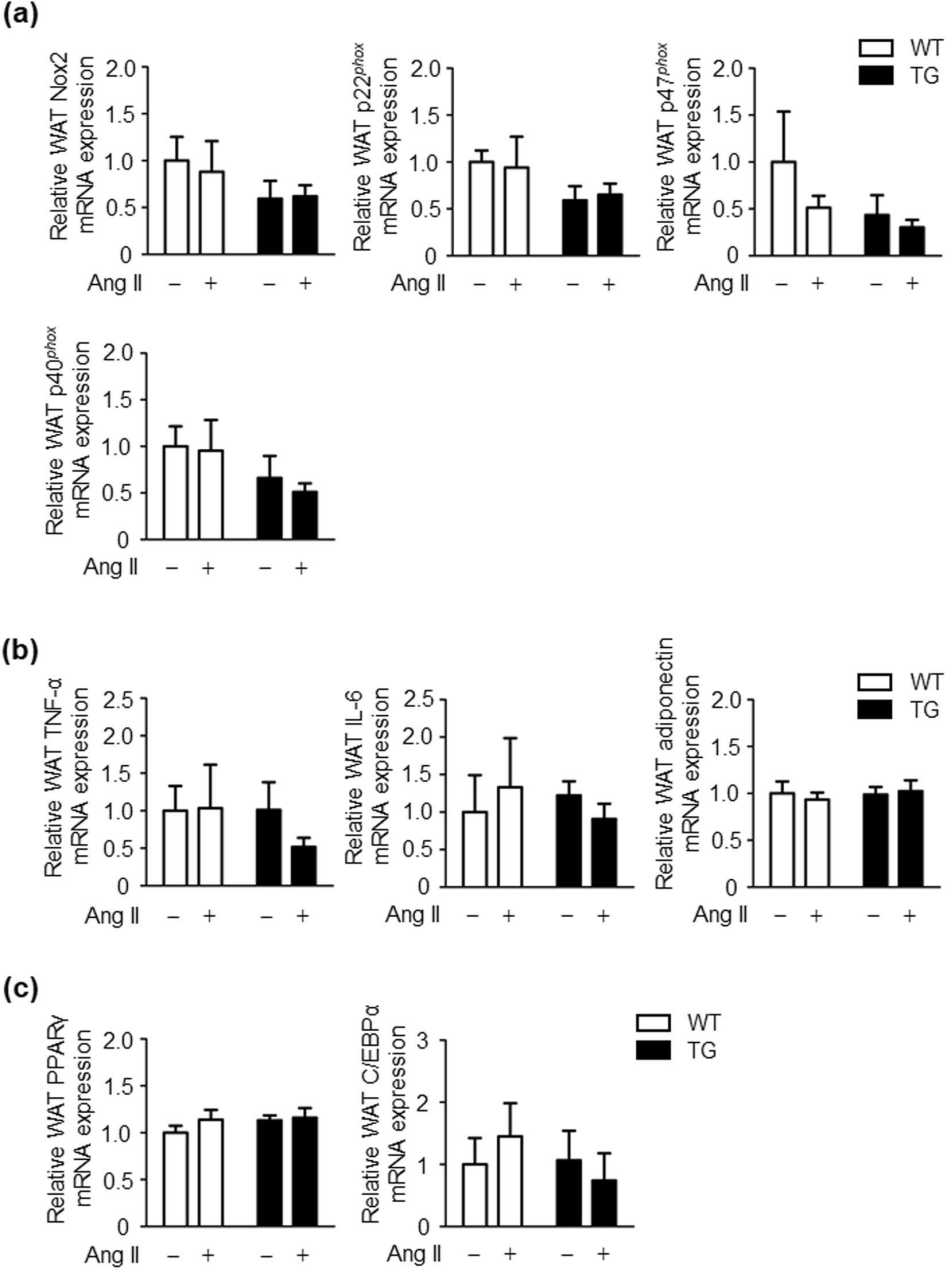
Ang II infusion does not affect oxidative stress in adipose tissue, adipokines, and adipocyte differentiation in WT and TG mice. (**a**) Relative NOX2, p22^*phox*^, p47^*phox*^, and p40^*phox*^ mRNA expression levels in WAT of WT and TG mice after 2 weeks of vehicle or Ang II infusion. Values are expressed as mean ± SE (*n* = 5 in each group). (**b**) Relative tumour necrosis factor-α, interleukin-6, and adiponectin mRNA expression levels in WAT of WT and TG mice after 2 weeks of vehicle or Ang II infusion. Values are expressed as mean ± SE (*n* = 5 in each group). (**c**) Relative PPARγ and C/EBPα mRNA expression levels in WAT of WT and TG mice after 2 weeks of vehicle or Ang II infusion. Values are expressed as mean ± SE (*n* = 5 in each group). WT, wild-type; TG, ATRAP transgenic mice; Ang II, angiotensin II.

To investigate the mechanisms involved in the inhibition of insulin resistance in response to chronic Ang II infusion in TG mice, we examined nicotinamide adenine dinucleotide phosphate (NADPH) oxidase expression in WAT from WT and TG mice. NADPH oxidase–derived reactive oxygen species (ROS) function as important intracellular second messengers to impair insulin signalling in adipose tissue^17,18^. There were no significant differences in NADPH oxidase components in WAT between the vehicle- and Ang II-infused groups in WT and TG mice (Fig. 4a). We next investigated the function of adipose tissue as an endocrine organ for releasing adipokines. The mRNA expression of tumor necrosis factor-α (TNF-α), interleukin-6 (IL-6) and adiponectin in WAT was comparable in the vehicle- and Ang II- infused groups in WT and TG mice (Fig. 4b). There was also no significant difference in mRNA expression of adipokines in WAT between WT and TG mice, regardless of the presence or absence of Ang II (Fig. 4b). Ang II reportedly impairs adipogenic differentiation of preadipose cells^19^. Therefore, we examined mRNA expression of peroxisome proliferator-activated receptor γ (PPARγ) and CCAAT enhancer-binding protein α (C/EBPα), which are critical transcription factors^20^, in WAT from WT and TG mice. Ang II infusion did not affect mRNA PPARγ and C/EBPα expression in adipose in either WT or TG mice (Fig. 4c). These results indicate that inhibition of Ang II-induced insulin resistance in TG mice is not mediated by suppression of AT1R signalling in adipose tissue.

### Effects of Ang II infusion on oxidative stress in skeletal muscle tissue in WT and TG mice

**Figure 5:**
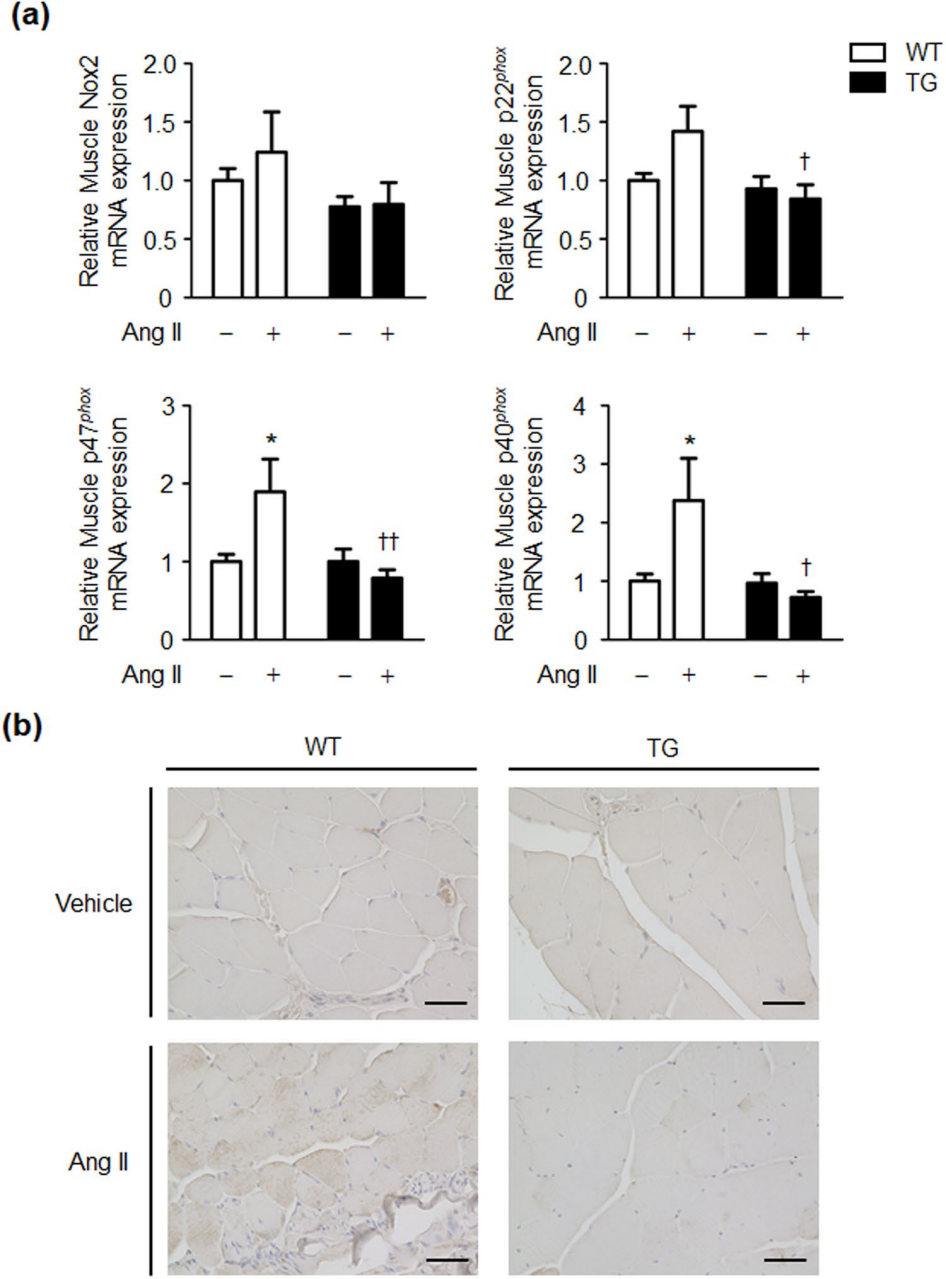
Ang II-induced enhancement of NADPH oxidases and ROS is suppressed in skeletal muscle tissue of TG mice. (**a**) Relative NOX2, p22phox, p47phox, and p40phox mRNA expression levels in skeletal muscle tissue of WT and TG mice after 2 weeks of vehicle or Ang II infusion. (**b**) Representative images of immunohistochemical staining for 4-HNE in skeletal muscle tissue of WT and TG mice after 2 weeks of vehicle or Ang II infusion (magnification, ×400; scale bar = 100 μm). Values are expressed as mean ± SE (*n* = 5 in each group) *P < 0.05, vs vehicle; ^†^*P* < 0.05, ^††^*P* < 0.01, vs WT mice. WT, wild-type; TG, ATRAP transgenic mice; Ang II, angiotensin II.

Skeletal muscle tissue is important as a major target organ for insulin^21,22^. We next examined mRNA expression of the NADPH oxidase components (NOX2, p22^*phox*^, p47^*phox*^, and p40^*phox*^) in skeletal muscle tissue of WT and TG mice. mRNA expression levels of p47^*phox*^ and p40^*phox*^ in skeletal muscle tissue were significantly higher after Ang II infusion compared with vehicle infusion in WT mice (Fig. 5a). However, the Ang II-induced increase in p47^*phox*^ and p40^*phox*^ mRNA expression in skeletal muscle tissue was completely suppressed in TG mice (Fig. 5a). To further evaluate the changes in ROS status in skeletal muscle tissue of WT and TG mice, we examined immunostaining of 4-hydroxy-2-nonenal (4-HNE), which reflects tissue ROS generation in tissue^23,24^. Immunostaining of 4-HNE in skeletal muscle tissue was enhanced after Ang II infusion compared with vehicle infusion in WT mice (Fig. 5b). However, the Ang II-induced increase in 4-NHE immunostaining was not observed in skeletal muscle tissue of TG mice (Fig. 5b). These results indicate that the Ang II-induced increase in several NADPH oxidase components and ROS generation are inhibited in skeletal muscle tissue of TG mice.

### Effects of Ang II infusion on Akt signalling and glucose transporter type 4 expression in skeletal muscle of WT and TG mice

**Figure 6:**
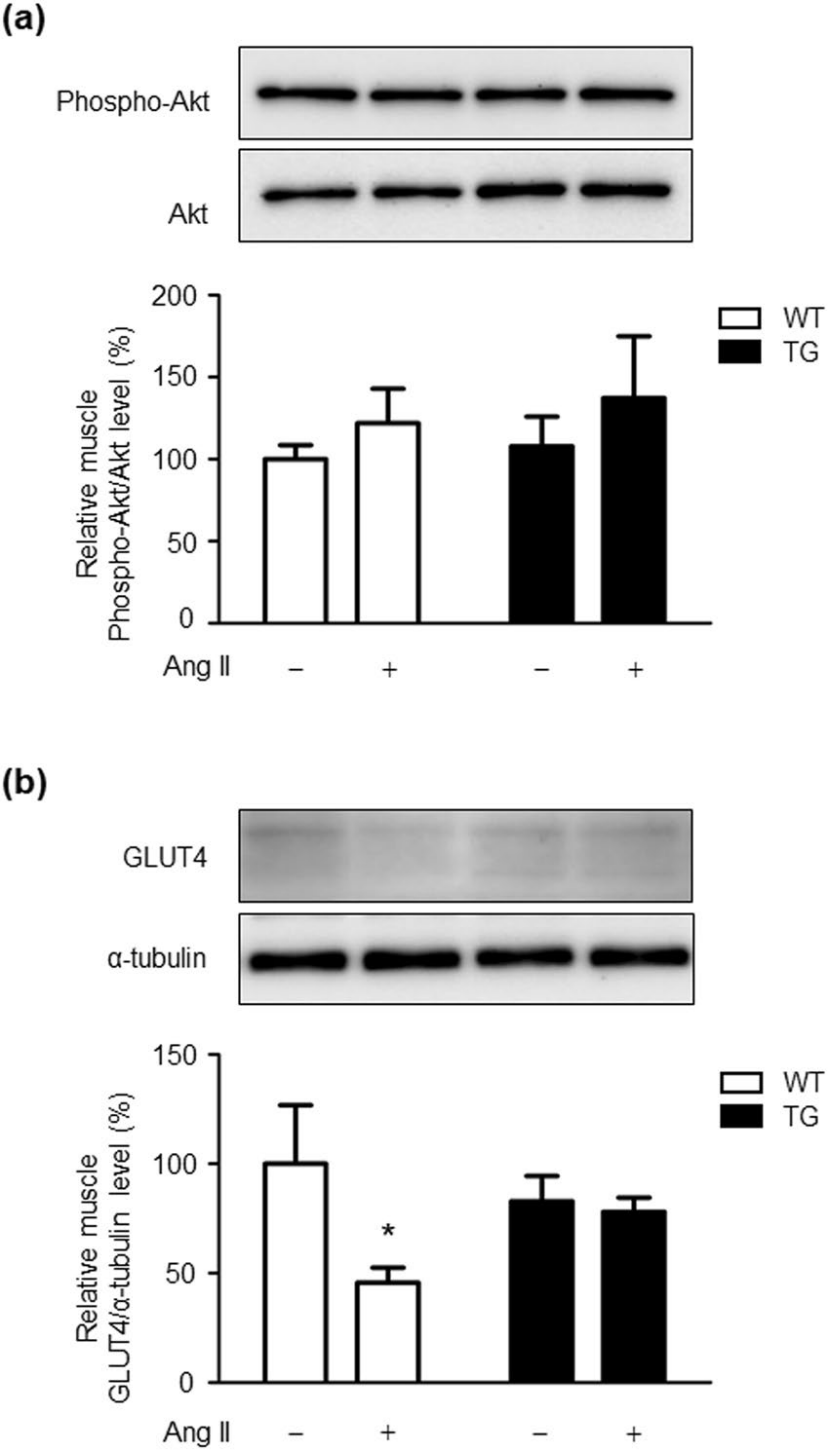
Effects of Ang II infusion on Akt activation and GLUT4 expression in skeletal muscle tissue of WT and TG mice. Representative western blots and quantitative analysis of phospho-Akt and Akt (**a**), and GLUT4 (**b**) protein levels in skeletal muscle from WT and TG mice after 2 weeks of vehicle or Ang II infusion. Values are expressed as mean ± SE (*n* = 5–8 in each group) *P < 0.05, vs vehicle. WT, wild-type; TG, ATRAP transgenic mice; Ang II, angiotensin II; GLUT4, glucose transporter 4.

We further investigated the mechanism of insulin resistance caused by skeletal muscle tissue NADPH oxidase–derived ROS generation in Ang II-infused mice. We examined protein levels of phospho-Akt and glucose transporter type 4 (GLUT4) in skeletal muscle tissue. There was no significant difference in phospho-Akt in skeletal muscle tissue between the vehicle- and Ang II-infused groups in WT and TG mice (Fig. 6a). However, GLUT4 expression was significantly lower after Ang II infusion compared with vehicle infusion in skeletal muscle tissue of WT mice (Fig. 6b). This Ang II-induced downregulation of GLUT4 expression was inhibited in skeletal muscle tissue of TG mice.

### Effects of Ang II infusion on skeletal muscle p38 mitogen-activated protein kinase activation in WT and TG mice

**Figure 7:**
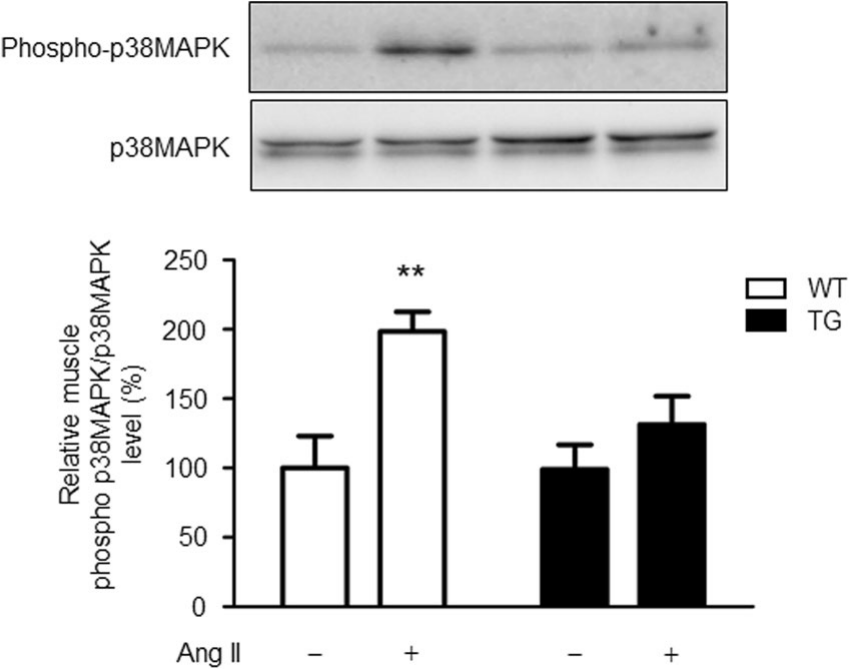
Ang II-induced activation of p38 MAPK is suppressed in skeletal muscle tissue of TG mice. Representative western blots and quantitative analysis of phospho-p38 MAPK and p38 MAPK protein levels in skeletal muscle tissue from WT and TG mice after 2 weeks of vehicle or Ang II infusion. Values are expressed as mean ± SE (*n* = 5 in each group) **P < 0.01, vs vehicle. WT, wild-type; TG, ATRAP transgenic mice; Ang II, angiotensin II; MAPK, mitogen-activated protein kinase.

As the downstream signalling pathway of the AT1 receptor, we compared the activation of mitogen-activated protein kinase (MAPK) in response to Ang II infusion in skeletal muscle tissue of WT and TG mice. Skeletal muscle tissue p38 MAPK was significantly activated after 2 weeks of Ang II infusion in WT mice (Fig. 7). However, Ang II-induced activation of p38 MAPK was completely suppressed in skeletal muscle tissue of TG mice. These results indicate that suppression of over-activated AT1 receptor signalling by ATRAP enhancement in skeletal muscle tissue contributes to inhibition of Ang II-induced insulin resistance in TG mice.

## Discussion

ATRAP TG mice used in the present study were generated using a 5.4-kb adiponectin promoter, which has recently been reported to have high specific gene tractability for adipose tissue^14,25–27^. Although the adipose tissue is the main tissue in which ATRAP is highly expressed, we found that there is also significantly higher ATRAP expression in skeletal muscle tissue in TG mice (approximately 1.6-fold higher expression than WT mice at the mRNA level). Adiponectin in the blood stream is mainly secreted by adipocytes, but several studies have indicated that endogenous adiponectin is also produced by skeletal muscle tissues. In addition, adiponectin production in skeletal muscle tissue is increased in response to metabolic stress, such as that induced by a high-fat diet^28–30^. The finding that there is a slight increase in ATRAP expression in skeletal muscle tissue of TG mice compared with that of WT mice is consistent with previous knowledge regarding ATRAP.

We assessed physiological parameters of WT mice that were affected by administration of a low dose of Ang II (100 ng/kg/min) over 2 weeks. We found that although there were no significant changes in body weight and blood pressure, there was an increase in non-fasting plasma insulin levels. In general, administration of Ang II results in elevated blood pressure, and causes muscle atrophy and cachexia, among other effects^31,32^. With respect to animal species differences in susceptibility to Ang II treatment, rats exhibit susceptibility to progressive hypertension in response to Ang II infusion, whereas mice are relatively resistant to Ang II-induced hypertension^7,33–35^. In the present study, 100 ng/kg/min doses of Ang II infusion into mice did not cause elevated blood pressure and weight loss, which is consistent with the previous reports^33,34^. However, administration of subpressor doses of Ang II promoted insulin resistance as estimated by the ITT. Additionally, Ang II stimulus tended to cause impaired glucose tolerance as estimated by the GTT, although this did not reach statistical significance.

There are several reports that chronic administration of low doses of Ang II promotes insulin resistance in mice and rats^7,9^. Studies on the mechanism of this promotion have reported that an increase in oxidative stress by activation of NADPH oxidase through skeletal muscle tissue AT1 receptor, disorders of GLUT4 expression and translocation in the insulin downstream pathway through activation of MAPK resulting from increased oxidative stress^36,37^, and suppression of Akt phosphorylation are involved^38,39^. In our study, Ang II infusion decreased GLUT4 expression, but did not affect Akt activation in skeletal muscle tissue of WT mice. This finding is partially consistent with previous reports that chronic Ang II infusion induces insulin resistance without decreasing Akt activation in mice and rats^7,9^. Although administration of low doses of Ang II caused activation of the p47^*phox*^/p40^*phox*^-ROS-p38MAPK pathway and decreased GLUT4 expression in skeletal muscle tissue of WT mice, this change was completely suppressed in skeletal muscle tissue of TG mice. Collectively, these findings indicate that increased ATRAP expression in skeletal muscle tissue suppresses hyperactivation of AT1 receptor signalling^40,41^.

We recently conducted an investigation on the effect of a high-fat diet on the same type of ATRAP transgenic mice that were used in the present study^15,42^. We found that enhancement of adipose ATRAP expression suppressed oxidative stress and phosphorylation of p38 MAPK in response to the high-fat diet in adipose tissue. Additionally, enhancement of adipose ATRAP expression improved dysfunction of adipokine regulation and inhibited obesity-related metabolic disorders^15,42^. However, in the present study, enhancement of adipose ATRAP expression did not show a protective effect on the adipose tissue profile. This finding is probably related to the fact that administration of Ang II had no effect on oxidative stress and adipokine levels in adipose tissue of WT mice. Therefore, insulin resistance due to a direct effect of Ang II stimulation, unrelated to dietary obesity, might primarily occur in skeletal muscle tissue. As a result, adipose tissue has almost no involvement in formation of this pathological condition.

The following limitations of this study need to be considered. First, the TG mouse model is characterised by a high ATRAP expression level in adipose tissue. Therefore, there needs to be confirmation that the same results can be obtained when using a model in which a high ATRAP expression level is specific to skeletal muscle tissue^43^. If such transgenic mice can be generated, the possibility must be kept in mind that an increase in ATRAP expression levels only in skeletal muscle tissue may cause major deviations above physiological levels^44,45^. Our finding that increased ATRAP expression levels in skeletal muscle tissue just under 2-fold completely blocked insulin resistance caused by Ang II treatment suggests the powerful beneficial effect of ATRAP in skeletal muscle tissue. Second, ATRAP and AT1 receptor expression in skeletal muscle cells isolated from skeletal muscle tissue was not examined in the present study. A possibility that other non-skeletal muscle cells in skeletal muscle tissue or skeletal muscle blood flow might be related to formation of insulin resistance provoked by Ang II infusion cannot be excluded completely. Therefore, further studies will be needed to investigate expression and function of ATRAP and AT1 receptor in skeletal muscle cells isolated from skeletal muscle tissue between WT and TG mice.

In conclusion, chronic administration of low-dose Ang II in WT mice causes increased oxidative stress in skeletal muscle tissue and insulin resistance, but there is no effect on the adipose tissue profile. This Ang II-induced insulin resistance is suppressed by increased ATRAP expression levels in skeletal muscle tissue. Our findings suggest that even in patients who have not yet presented with obesity or hypertension, hyperactivity of AT1 receptor signalling is related to formation of pathological insulin resistance related to metabolic syndrome. In addition, ATRAP activation therapy in insulin target organs (e.g., adipose tissue and skeletal muscle tissue) may be widely applied for treating lifestyle diseases that are not accompanied by obesity or hypertension.

## Methods

### Animals

The mice were housed in a controlled environment with a 12-hour light–dark cycle at a temperature of 25 °C. The mice were allowed free access to food and water. Mice were fed a standard diet (0.3% NaCl, 3.6 kcal/g, and 13.3% energy as fat; Oriental MF, Oriental Yeast Co, Ltd.). This study was performed in accordance with the National Institute of Health guidelines for the use of experimental animals. All animal studies were reviewed and approved by the Animal Studies Committee of Yokohama City University.

### Generation of TG mice overexpressing ATRAP in adipose tissue and skeletal muscle tissue

TG mice overexpressing ATRAP in adipose tissue and skeletal muscle tissue were generated on a C57BL/6 J background, as previously described^15^. Briefly, hemagglutinin-tagged mouse ATRAP (HA-ATRAP) cDNA was subcloned into a transgenic vector between the 5.4-kb fragment of the adiponectin promoter (kindly provided by Dr. Philipp E. Scherer, The University of Texas Southwestern Medical Center) and 3′ untranslated region (3′UTR). This construct was microinjected into the pronucleus of fertilised mouse embryos. TG mice were identified by PCR using forward (TGCTTGGGGCAACTTCACTATC) and reverse (ACGGTGCATGTGGTAGACGAG) primers. Mice were mated with C57BL/6 mice to obtain TG mice and their littermate WT mice for the experiments.

### Ang II treatment

Ang II infusion was performed as previously described^46^. Briefly, Ang II (100 ng/kg/min) was infused subcutaneously in male WT and TG mice (9–11 weeks old) for 14 days using an osmotic minipump (Model 2002; ALZET, Palo Alto, CA, USA).

### Blood pressure measurement

Systolic blood pressure and heart rate were measured by the tail-cuff method (BP-Monitor MK-2000; Muromachi Kikai Co.). The MK-2000 monitor enabled measurement of blood pressure without preheating the animals and using anaesthesia. This procedure avoided this stressful condition, as described previously^15^. All measurements were performed between 10:00–14:00 hours. At least eight readings were taken for each measurement.

### Biochemical assays

Blood samples were obtained by cardiac puncture when the mice were sacrificed in the fed state. Whole blood samples were centrifuged at 3000 rpm at 4 °C for 15 minutes to separate the plasma. Enzymatic assay kits were used for determining plasma glucose, free fatty acid, triglyceride, and total cholesterol levels (Wako Pure Chemical). Plasma insulin concentrations were measured with a commercially available ELISA kit (The Morinaga Institute of Biological Science, Inc.).

### GTT and ITT

The GTT and ITT were performed as previously described^15,47^. Briefly, for the GTT, blood glucose concentrations were measured with a blood glucose test metre (Glutest Neo Super; Sanwa-Kagaku) using blood samples that were taken from the tail tip of overnight-fasted mice. Samples were taken at baseline and at 15, 30, 60, and 120 minutes after intraperitoneal injection of glucose (1 g/kg body weight). For the ITT, insulin (0.75 U/kg body weight in 0.1% BSA; Humulin R-Insulin; EliLilly & Co.) was administered via intraperitoneal injection after 4-hour fasting. Blood glucose concentrations were measured at baseline and at 15, 30, 60, and 120 minutes after the injection.

### Real-time quantitative RT-PCR analysis

Total RNAs were extracted from WAT, interscapular BAT, and soleus muscle tissue with ISOGEN (Nippon Gene), and the cDNA was synthesized using the SuperScript III First-Strand System (Invitrogen). Real-time quantitative RT-PCR was performed with an ABI PRISM 7000 Sequence Detection System by incubating the reverse transcription product with TaqMan PCR Master Mix and a designed Taqman probe (Applied Biosystems), essentially as described previously^47^. The mRNA levels were normalized to those of the 18SrRNA or glyceraldehyde-3-phosphate dehydrogenase (GAPDH) control.

### Immunoblot analysis

Immunoblot analysis was performed as described previously^15,46^. Briefly, total protein extract was prepared from tissues with sodium dodecyl sulfate-containing sample buffer. The protein concentration of each sample was measured with a Detergent Compatible Protein Assay Kit (Bio-Rad) using bovine serum albumin as the standard. Equal amounts of protein extract from tissue samples were fractionated on a 5–20% polyacrylamide gel (ATTO). The gel was then transferred to a polyvinylidene difluoride membrane using the iBlot Dry Blotting System (Invitrogen). Membranes were blocked for 1 hour at room temperature with phosphate-buffered saline containing 5% skim milk powder. Membranes were probed overnight at 4 °C with specific primary antibodies to GLUT4 (Abcam), Akt, phospho-Akt (Cell Signaling Technology), p38 MAPK (Santa Cruz Biotechnology), and phospho-p38 MAPK (Promega Corporation). Membranes were washed and further incubated with secondary antibodies for 15 minutes at room temperature. The sites of the antibody–antigen reaction were visualised by enhanced chemiluminescence substrate (GE Healthcare). Images were analysed quantitatively using a Fuji LAS-3000 image analyser (Fuji Film). For analysis of phospho-Akt, tissues were collected as described previously^15^. Briefly, after 4-hour fasting, mice were deeply anesthetized with an intraperitoneal injection of pentobarbital. Insulin (5 U/kg body weight in 0.1% BSA; Humulin R-Insulin; Eli Lilly & Co.) was injected through the inferior vena cava, and tissues were quickly collected 2 minutes after the injection.

### Immunohistochemical analysis

Immunohistochemical analysis was performed as described previously^24,48^. Soleus muscle tissue from mice was fixed with 4% paraformaldehyde and subsequently embedded in paraffin. Sections of 4-μm thickness were dewaxed and rehydrated, and antigen retrieval was performed by microwave heating. The sections were blocked for endogenous biotin activity using peroxidase blocking reagent (DAKO) and treated for 60 minutes with 10% normal goat serum in phosphate-buffered saline. The sections were then incubated with anti-4-HNE antibody diluted to 1:100 (Abcam), anti-myoglobin antibody (Nichirei Biosciences), and anti-ATRAP antibody diluted to 1:100.

### Statistical analysis

Data are expressed as mean ± SE. Differences were analysed as follows. 2-factor ANOVA with Bonferroni posttests was used to test for differences in Ang II infusion and genotype (Figs 2d, 4a and b, 5a, 6a and b and 7); Unpaired t-test was used to test for differences in WT vs TG mice (Fig. 1a) or differences in vehicle vs Ang II infusion within each genotype (Table and Fig. 3a–h); Repeated measures ANOVA was used to test for differences over time (Fig. 2a–c). A *P* value < 0.05 was considered statistically significant.

## Electronic supplementary material

Supplementary Information
