# Supplementary material for: Angiotensin II Type 1 Receptor-associated Protein Inhibits Angiotensin II-induced Insulin Resistance with Suppression of Oxidative Stress in Skeletal Muscle Tissue
Source: Sci Rep. 2018 Feb 12;8:2846. doi: 10.1038/s41598-018-21270-8 (PMC5809432; doi:10.1038/s41598-018-21270-8)

**Supplementary information**

**Angiotensin II Type 1 Receptor-associated Protein Inhibits Angiotensin II-induced Insulin Resistance with Suppression of Oxidative Stress in Skeletal Muscle Tissue**

Running title:

Muscle ATRAP Inhibits Angiotensin II-induced Insulin Resistance

Kohji Ohki, MD;^†,1^ Hiromichi Wakui, MD, PhD;^†,1^ Nozomu Kishio;^†,1^ Kengo Azushima, MD, PhD;^1,2^ Kazushi Uneda, MD, PhD;^1^ Sona Haku, MD;^1^ Ryu Kobayashi, MD, PhD;^1^ Kotaro Haruhara, MD;^1^ Sho Kinguchi, MD;^1^ Takahiro Yamaji, MD;^1^ Takayuki Yamada, MD;^1^ Shintaro Minegishi, MD, PhD;^1^ Tomoaki Ishigami, MD, PhD;^1^ Yoshiyuki Toya, MD, PhD;^1^ Akio Yamashita, PhD;^3^ Kento Imajo, MD, PhD;^4^ Atsushi Nakajima, MD, PhD;^4^ Ikuma Kato, MD, PhD;^5^ Kenichi Ohashi, MD, PhD;^5^ Kouichi Tamura, MD, PhD^1^

^†^ These authors contributed equally to this work.

^1^Department of Medical Science and Cardiorenal Medicine, Yokohama City University Graduate School of Medicine, Yokohama, Japan; ^2^Cardiovascular and Metabolic Disorders Program, Duke-NUS Medical School, Singapore; ^3^Department of Molecular Biology, Yokohama City University Graduate School of Medicine, Yokohama, Japan; ^4^Department of Gastroenterology and Hepatology, Yokohama City University Graduate School of Medicine, Yokohama, Japan; ^5^Department of Molecular Pathology, Yokohama City University Graduate School of Medicine.

Correspondence to:

Hiromichi Wakui, M.D., Ph.D., or Kengo Azushima, M.D., Ph.D. or Kouichi Tamura, M.D., Ph.D., F.A.C.P., F.A.H.A.

Department of Medical Science and Cardiorenal Medicine

Yokohama City University Graduate School of Medicine

3-9 Fukuura, Kanazawa-ku, Yokohama 236-0004, Japan.

Tel: 81-45-787-2635 Fax: 81-45-701-3738

E-mail: [hiro1234@yokohama-cu.ac.jp](mailto:hiro1234@yokohama-cu.ac.jp) or [azushima@yokohama-cu.ac.jp](mailto:azushima@yokohama-cu.ac.jp) or [tamukou@med.yokohama-cu.ac.jp](mailto:tamukou@med.yokohama-cu.ac.jp)


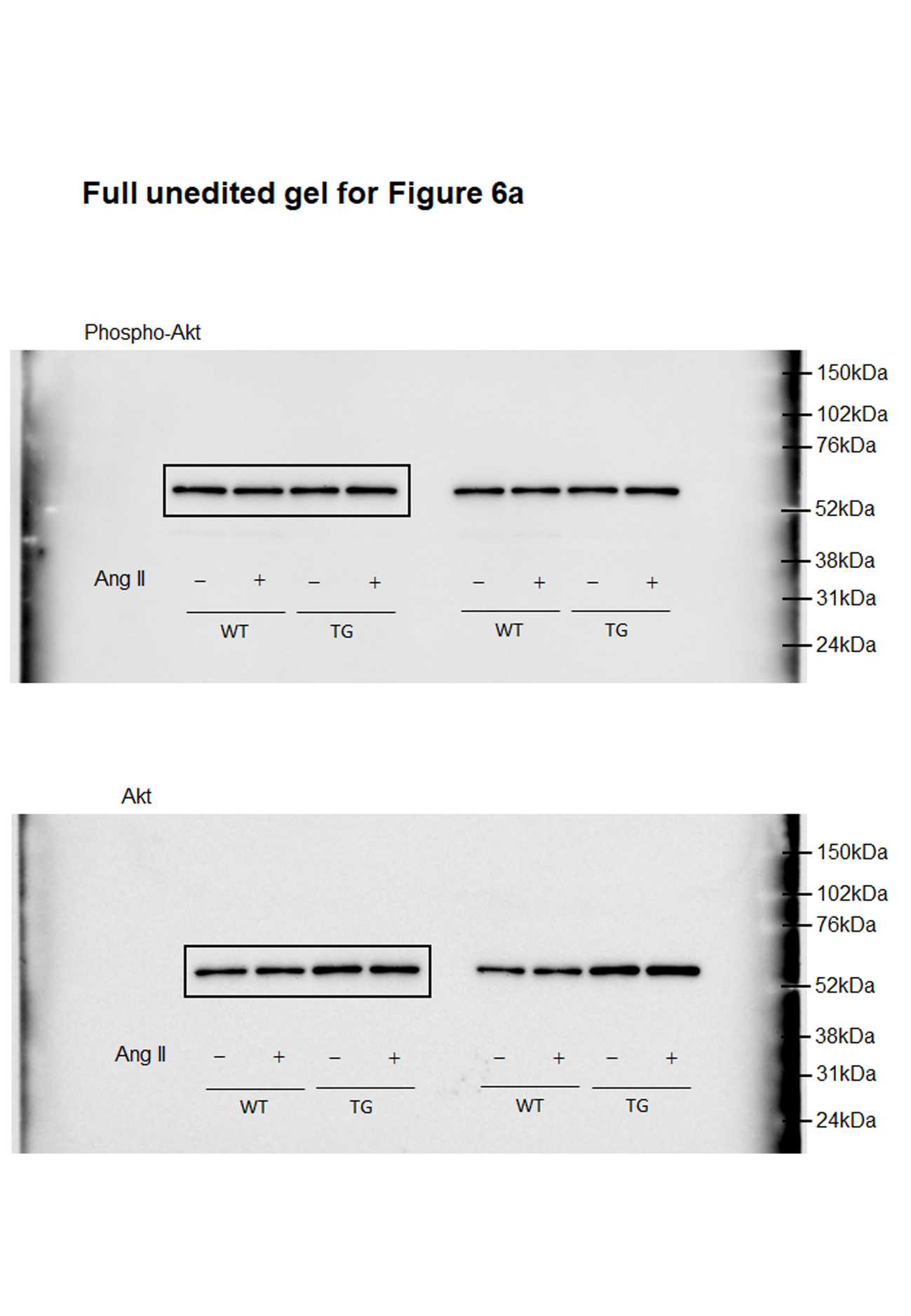


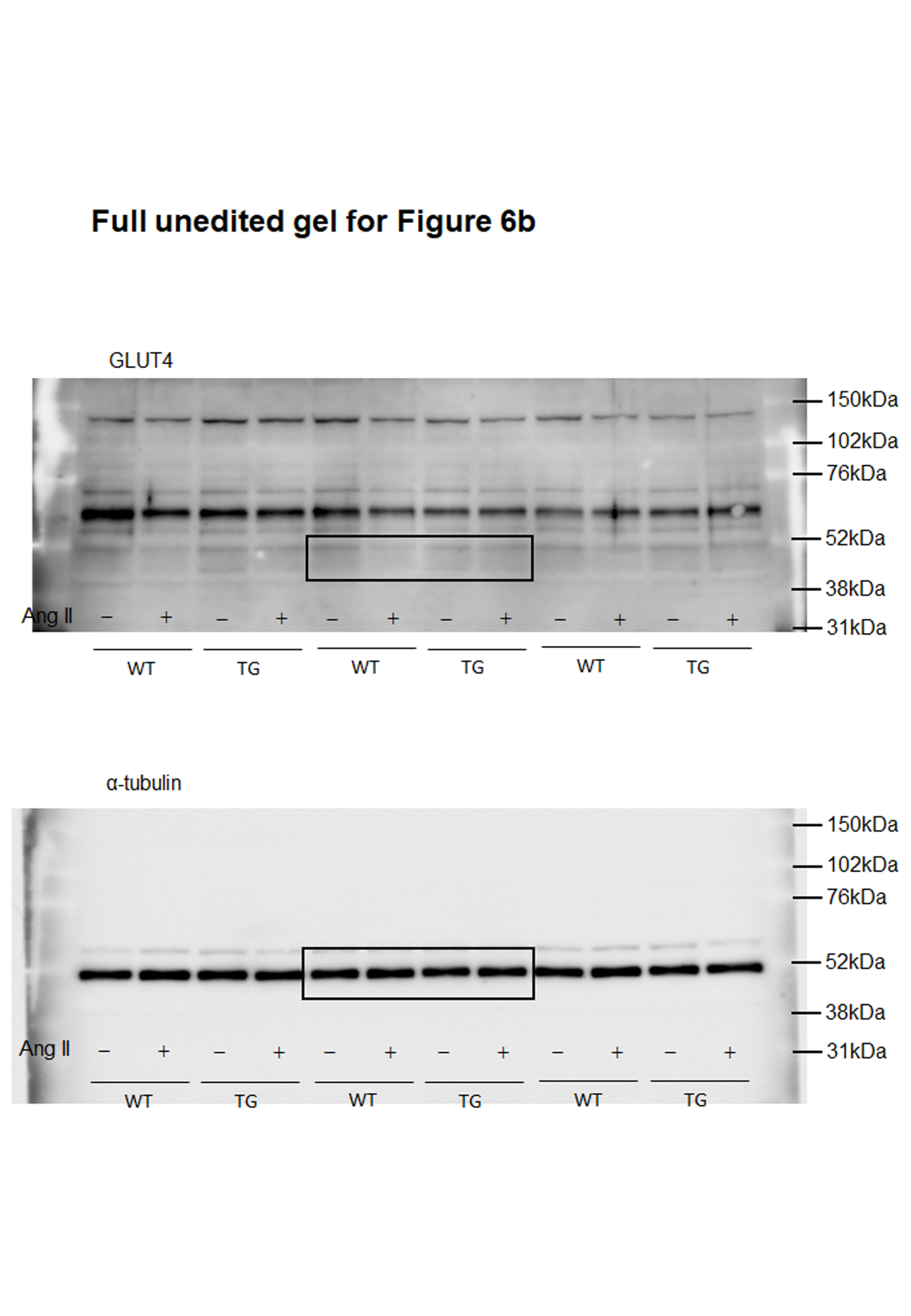


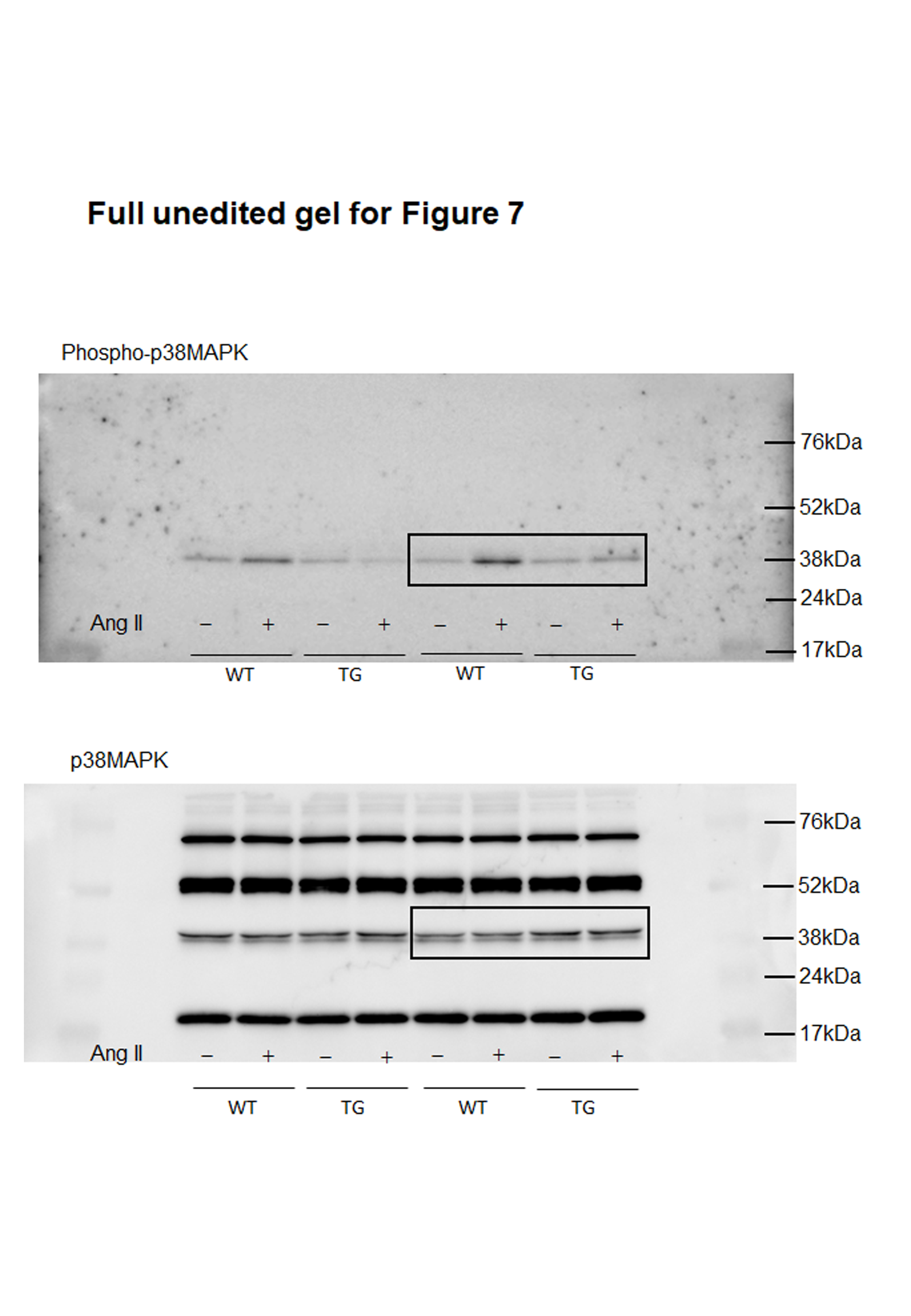

Supplement: Supplementary file 1 — Supplementary Information [file 41598_2018_21270_MOESM1_ESM.doc]
